# Effects of Concurrent Training on Biomarkers, Morphological Variables, and Physical Performance in People with Sarcopenic Obesity: A Meta-Analysis with Meta-Regression

**DOI:** 10.3390/medicina61091697

**Published:** 2025-09-18

**Authors:** Jordan Hernandez-Martinez, Edgar Vásquez-Carrasco, Izham Cid-Calfucura, Cristian Sandoval, Tomás Herrera-Valenzuela, Cristian Núñez-Espinosa, Braulio Henrique Magnani Branco, Pablo Valdés-Badilla

**Affiliations:** 1 Department of Physical Activity Sciences, Universidad de Los Lagos, Osorno 5290000, Chile; jordan.hernandez@ulagos.cl; 2 Department of Education, Faculty of Humanities, Universidad de la Serena, La Serena 1700000, Chile; 3 School of Occupational Therapy, Faculty of Psychology, Universidad de Talca, Talca 3465548, Chile; edgar.vasquez@utalca.cl; 4 Centro de Investigación en Ciencias Cognitivas, Faculty of Psychology, Universidad de Talca, Talca 3465548, Chile; 5 Department of Physical Activity, Sports and Health Sciences, Faculty of Medical Sciences, Universidad de Santiago de Chile (USACH), Santiago 8370003, Chile; izham.cid@gmail.com (I.C.-C.); tomas.herrera@usach.cl (T.H.-V.); 6 Escuela de Tecnología Médica, Facultad de Salud, Universidad Santo Tomás, Los Carreras 753, Osorno 5310431, Chile; cristian.sandoval@ufrontera.cl; 7 Departamento de Medicina Interna, Facultad de Medicina, Universidad de La Frontera, Temuco 4811230, Chile; 8 Escuela de Medicina, Universidad de Magallanes, Punta Arenas 6200000, Chile; cristian.nunez@umag.cl; 9 Centro Asistencial Docente e Investigación, Universidad de Magallanes, Punta Arenas 6200000, Chile; 10 Graduate Program in Health Promotion, Cesumar University (UniCesumar), Maringá 87050-900, Brazil; braulio.branco@unicesumar.edu.br; 11 Department of Physical Activity Sciences, Faculty of Education Sciences, Universidad Católica del Maule, Talca 3530000, Chile; 12 Sports Coach Career, Faculty of Life Sciences, Universidad Viña del Mar, Viña del Mar 2520000, Chile

**Keywords:** abdominal fat, body fat, laboratory markers, physical therapists, muscle strength, physical fitness

## Abstract

*Background and Objectives:* This systematic review and meta-analysis aimed to analyze the available body of published peer-reviewed randomized controlled trials (RCTs) on the effects of concurrent training (CT) on biomarkers, morphological variables, and physical performance in people with sarcopenic obesity. *Materials and Methods:* Using six databases—PubMed, Medline, CINAHL Complete, Scopus, Cochrane Library, and Web of Science—a comprehensive literature search was conducted through July 2025. The GRADE, TESTEX, Rob 2, and PRISMA tools were used to assess the methodological quality and certainty. The protocol was registered in PROSPERO (CRD420251052935). *Results*: Out of 669 records, 8 RCTs with a total of 453 participants (68.9 ± 11.1 years) were included. Fifteen overall and three subgroup meta-analyses revealed significant improvements (*p* < 0.05) in insulin-like growth factor-1 (ES = 1.01, 95% CI = 0.26 to 1.75, *p* = 0.008) and leptin (ES = 2.54, 95% CI = 0.07 to 5.01, *p* = 0.04) levels; significant decreases (*p* < 0.05) in body mass index (ES = 0.54, 95% CI = 0.12 to 0.97, *p* = 0.01), waist circumference (ES = 1.80, 95% CI = 0.32 to 2.12, *p* = 0.008), and body fat (BF, ES = 1.31, 95% CI = 0.53 to 2.09, *p* = 0.001); and significantly increased (*p* < 0.05) appendicular skeletal muscle mass/weight (ES = 0.42, 95% CI = 0.14 to 0.71, *p* = 0.004), walking speed (ES = 1.80, 95% CI = 1.05 to 2.55, *p* = 0.000), and knee extension (ES = 0.76, 95% CI = 0.09 to 1.42, *p* = 0.02). However, no significant improvements (*p* > 0.05) were observed in IL-6, CRP, total cholesterol, triglycerides, trunk fat, BF mass, and MIHS. On the other hand, an important result in the meta-regression revealed that weeks of training can predict decreases in BF (R^2^ = 0.32; *p* = 0.02). *Conclusions*: CT has been associated with significant clinical improvements in biomarkers related to increased muscle mass and decreased BF percentage.

## 1. Introduction

After 40 years of age, there are natural changes in morphological variables, decreasing muscle mass accompanied by an increase in body fat, affecting both males and females [1]. However, when these changes in body composition are accompanied by alterations in physical function, mainly muscle strength, such as walking speed, they produce a geriatric syndrome called sarcopenic obesity, as indicated in the ESPEN and EASO Consensus Statement [2]. This excess body fat causes adipose tissue infiltration by adipocytes or macrophages, producing proinflammatory cytokines such as interleukin-6 (IL-6) and/or tumor necrosis factor alpha (TNF-α), negatively affecting muscle mass and strength [3], accompanied by accelerated muscle loss due to a symbiotic relationship between protein synthesis and degradation [3], leading to a decrease in the quality, quantity, and/or distribution of muscle fibers, causing specific atrophy of type II muscle fibers and a decrease in satellite cell reserves [3], affecting physical function and quality of life in people with this geriatric syndrome [4].

Therefore, it is important to carry out therapies that aim to improve these indicators in people with sarcopenic obesity [5], as they are effective non-pharmacological alternatives with a high impact on the clinical field and physical activity practices [6]. The most clinically recommended therapies are strength training (ST); endurance training (ET); and/or a combination of both, called concurrent training (CT) [7]. A meta-analysis conducted by Khalafi et al. [8] indicates that CT, unlike ST or ET alone, offers synergistic benefits by simultaneously improving cardiorespiratory capacity and muscle strength, making it a viable strategy for improving these variables in aging. In the meta-analysis performed by da Silva Gonçalves et al. [9] in people with sarcopenic obesity, ST, ET, and CT were compared, revealing significant improvements (*p* < 0.01) in favor of ST in terms of a decrease in body fat percentage, together with an increase in maximal isometric handgrip strength (MIHS), whereas in knee extension, both ST and CT improved, as did the 30 s chair stand test, which improved only gait speed in CT. Similar results were reported by Tian et al. [10] in people with sarcopenic obesity; the authors compared physical exercise (ST, ET, and CT) with control groups and reported a significant reduction in favor of physical exercise in terms of body fat percentage and significant improvements in MIHS, gait speed, 30 s chair stand test, and the timed up-and-go (TUG) test. Significant improvements in biomarkers such as C-reactive protein, IL-6, total cholesterol, high-density lipoprotein (HDL), low-density lipoprotein (LDL), and triglycerides have not been reported.

Although previous studies have demonstrated the effects of CT on body composition, physical performance, and certain biomarkers [5,9], there remains a paucity of evidence regarding how specific training parameters—such as intervention duration, weekly frequency, and session length—influence these outcomes, particularly in individuals with sarcopenic obesity. Moreover, a more comprehensive evaluation of various biomarkers is warranted to elucidate their role in mediating responses to CT, especially in relation to alterations in body composition and functional capacity. Consequently, this meta-analysis aimed to evaluate the effects of CT on key biomarkers that have not been addressed in previous reviews (including IGF-1, IL-6, C-reactive protein, leptin, total cholesterol, and triglycerides), morphological variables, and physical performance in people with sarcopenic obesity. Additionally, subgroup analyses based on training dose (i.e., duration in weeks, session frequency, session duration, and total number of sessions) were conducted, along with meta-regressions, to determine whether training dose predicts variability in the observed outcomes. It is hypothesized that CT improves biomarkers related to increased muscle mass and reduced body fat, improving physical performance in people with sarcopenic obesity.

## 2. Methods

### 2.1. Protocol and Registration

The PRISMA guidelines were followed in this systematic review [11]. PROSPERO (the International Prospective Register of Systematic Reviews; ID code: CRD420251052935) has the protocol registered.

### 2.2. Eligibility Criteria

The inclusion criteria for this systematic review with meta-analysis were original peer-reviewed articles published until July 2025 that were unrestricted by language or publication date. The materials excluded were conference abstracts, books and book chapters, editorials, letters to the editor, protocol records, reviews, case studies, and trials. In addition, this systematic review used the population, intervention, comparator, outcome, and study design (PICOS) framework (see Table 1).

### 2.3. Information Search Process and Databases

The final literature search occurred on 15 July 2025, utilizing six primary electronic databases: PubMed, MEDLINE, CINAHL Complete, the Cochrane Library, Scopus, and Web of Science (Core Collection). The search strategy utilized Medical Subject Headings (MeSH) alongside pertinent free-text terms related to CT, biomarkers, morphological variables, and physical performance in individuals with sarcopenic obesity. The complete search string was (“sarcopenic obesity” OR “obesity” OR “sarcopenic”) AND (“concurrent training” OR “resistance training” OR “resistance exercise” OR “strength training” OR “aerobic training” OR “aerobic exercises” OR “endurance training” OR “endurance exercises”) AND (“body composition” OR “body fat” OR “fat-free mass” OR “fat mass” OR “muscle mass” OR “mass” OR “nutritional status” OR “bone mineral density” OR “skeletal muscle mass index”) AND (“biomarkers” OR “IGF-1” OR “IL-6” OR “leptin” OR “C-reactive protein” OR “cholesterol” OR “glycemia” OR “triglycerides”) AND (“functional independence” OR “functional dependence” OR “functional mobility” OR “health condition” OR “falls” OR “fall risk” OR “risk of fall” OR “falling risk” OR “static balance” OR “dynamic balance” OR “walking speed” OR “gait speed” OR “mobility” OR “strength” OR “muscle”).

Two independent subject-matter experts were consulted to review the selected studies and evaluate the inclusion and exclusion criteria, ensuring comprehensiveness. Experts needed to fulfill two eligibility criteria: (i) holding a PhD in Sport Sciences and (ii) having authored peer-reviewed publications on physical performance in various populations and/or related subjects in journals indexed by Journal Citation Reports^®^. The search strategy was withheld from the experts to mitigate potential bias in their assessment. A supplementary search was conducted on 15 July 2025 to identify any retractions or errata related to the included studies.

### 2.4. Study Selection and Data Collection Process

Study records were managed using the EndNote reference manager (version X9, Clarivate Analytics, Philadelphia, PA, USA). Two reviewers (J.H.-M. and I.C.-C.) independently conducted the literature searches, removed duplicates, screened titles and abstracts, and assessed the full-text articles for eligibility. No discrepancies were identified during this process. The same procedure was applied to studies identified through expert recommendations and manual searches of reference lists. Full-text articles deemed potentially relevant were thoroughly reviewed, and reasons for excluding studies that did not meet the inclusion criteria were documented.

### 2.5. Assessment of Methodological Quality

The methodological quality of the included studies was evaluated using the TESTEX scale, a validated tool specifically designed for exercise-based intervention trials [12]. The TESTEX tool allocates a maximum of 15 points—5 for study quality and 10 for reporting. Studies could be excluded based on low methodological quality as determined by this tool. Two reviewers (J.H.-M. and I.C.-C.) performed the assessment independently. In cases of disagreement or borderline scores, a third reviewer (T.H.-V.) acted as a referee, and further validation was provided by a fourth author (P.V.-B.) when necessary.

### 2.6. Data Synthesis

The subsequent data were extracted and analyzed from the studies included: (i) author and publication year; (ii) country of origin; (iii) study design; (iv) characteristics of participants, including sex and mean age; (v) type of intervention, sample size, and existence of a control group (CG); (vi) diagnostic criteria for sarcopenic obesity; (vii) methodology for assessing body composition; (viii) training volume (total intervention duration, weekly frequency, and session duration); (ix) training intensity; (x) biomarkers assessed; (xi) outcomes related to morphological variables; (xii) measures of physical performance; (xiii) reported adverse events; and (xiv) intervention adherence.

### 2.7. Assessment of Bias Risk

The risk of bias was assessed independently by two reviewers (J.H.-M. and E.V.-C.), utilizing the Cochrane Risk of Bias 2.0 (RoB 2) tool, which is specifically tailored for RCTs, in accordance with the guidelines presented in the Cochrane Handbook for Systematic Reviews of Interventions [13]. A third reviewer (P.V.-B.) evaluated and validated the assessments. The risk of bias was classified as “low,” “some concerns,” or “high,” according to five domains: the randomization process, deviations from intended interventions, missing outcome data, outcome measurement, and selection of the reported result.

### 2.8. Summary Measures for Meta-Analysis

According to established guidelines, meta-analyses were only performed when information from three or more separate trials was available for a particular outcome [14].

Hedges’ g was used to quantify effect sizes (ESs) for each outcome variable, specifically biomarkers, body composition, and physical performance, by comparing the means and standard deviations (SDs) of the CT and CG before and after the intervention. 95% confidence intervals (CIs) were utilized to report ES, and the standardized change score was employed. ES was interpreted as trivial (<0.2), small (0.2–0.6), moderate (>0.6–1.2), large (>1.2–2.0), very large (>2.0–4.0), and extremely large (>4.0). 

To account for anticipated heterogeneity among studies resulting from variations in intervention characteristics and demographics, a random-effects model (DerSimonian–Laird approach) was used. Body mass index, waist circumference, body fat percentage, appendicular skeletal muscle mass, trunk fat, fat mass, walking speed, MIHS, knee extension strength, IGF-1, IL-6, leptin, C-reactive protein, total cholesterol, and triglycerides (comparing CT vs. CG) were all measured, and their SMD and MD were calculated. According to the random-effects model, underlying variations in study populations or interventions cause genuine impact sizes to differ between studies.

Comprehensive Meta-Analysis Software (Version 2.0; Biostat, Englewood, NJ, USA) was used to perform meta-analyses. According to Verhagen et al. [15], *p* < 0.05 was the threshold for statistical significance. Cochran’s Q test [16] and the I^2^ statistic were used to evaluate heterogeneity across trials; the results were interpreted as low (<25%), moderate (25–50%), and high (>50%) heterogeneity [17].

Egger’s regression test [18] was used to evaluate publication bias and small-study effects. Visual examination of funnel plots (see Figures in Section 3.4 and Section 3.6) was also used.

### 2.9. Sensitivity Analyses

To assess the robustness and stability of the results, sensitivity analyses were conducted for all meta-analysis that included at least four studies. A leave-one-out approach was applied, whereby the pooled ES was recalculated after sequentially excluding each study, using the same DerSimonian–Laird random-effects model applied in the primary analysis. For each iteration, changes in the overall ES, statistical significance (*p*-value), and heterogeneity parameters (τ^2^, Q, and I^2^) were evaluated. In addition, targeted sensitivity analyses were performed by excluding studies identified as potentially problematic, based on two predefined criteria. (i) One criterion was a high risk of bias: studies classified as “high risk” in the overall assessment using the RoB 2.0 tool were excluded. This analysis was only performed when their removal left at least three studies in the meta-analysis. (ii) Outlier or influential studies were identified through a combination of visual inspection of forest plots and quantitative influence diagnostics [19]. A study was considered an outlier if its ES substantially deviated from the pooled confidence interval or if it exerted disproportionate influence on the overall estimate, as indicated by extreme values in one or more influence metrics. Specifically, the following thresholds were applied: DFBETAS > |1|, indicating substantial impact on model coefficients; Cook’s distance > 4/*n*, where *n* is the number of studies (values > 0.5 were also considered in small samples); and hat values > 2k/*n*, where k is the number of predictors (typically k = 1 in random-effects models). Studies exceeding one or more of these thresholds were flagged and excluded in additional sensitivity analyses to assess the robustness of the pooled effects.

### 2.10. Moderator Analyses

Using a random-effects model and independent single-factor analysis, potential sources of heterogeneity likely to influence the effects of training were selected a priori.

### 2.11. Subgroup Analyses

Since adaptive responses to CT programs can be affected by training dose (by weeks of training, sessions per week, minutes per session, and total sessions) [20], these factors were considered possible moderating variables.

### 2.12. Certainty of Evidence

Studies were categorized as having high, moderate, low, or very low confidence on the basis of their assessment of the GRADE scale [21]. Because studies with RCT designs were included, all analyses began with a high degree of certainty and were downgraded if concerns arose about bias, consistency, accuracy, precision, the directness of results, or the risk of publication bias [21]. Two authors evaluated the studies separately (J.H.-M., and I.C.-C.), and any disagreements were settled by agreement with a third author (E.V.-C.).

## 3. Results

**Figure 1:**
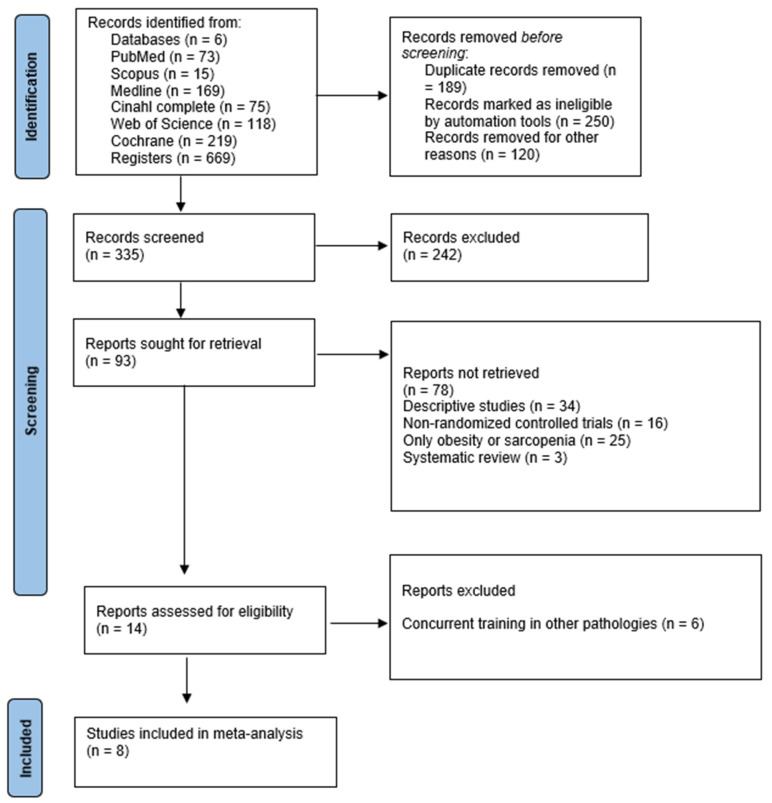
Flowchart of the review process. Legends: Based on the PRISMA guidelines according to Page, et al. [11].

Figure 1 details the flow diagram of the study selection process. A total of 669 records were identified through database searches. After removing duplicates, the titles, abstracts, and keywords were screened, yielding 335 potentially relevant references. In the subsequent analysis phase, 242 studies were excluded for not meeting the predefined inclusion criteria, leaving 93 articles for full-text review. Subsequently, 34 studies were excluded due to their descriptive design, 16 were non-randomized controlled trials, 25 focused solely on obesity or sarcopenia, and 3 were systematic reviews. Fourteen full-text articles were assessed for eligibility, of which six were excluded because they evaluated CT in populations with other pathologies not related to sarcopenic obesity. Ultimately, eight RCTs were included in the final qualitative and quantitative synthesis of this systematic review and meta-analysis [22,23,24,25,26,27,28,29].

### 3.1. Methodological Quality

The eight selected studies were analyzed via the TESTEX scale (Table 2). All the studies achieved a score equal to or greater than 60% on the scale [22,23,24,25,26,27,28,29], namely 11/15 [25], 12/15 [22,23,24,26,27,28], and 13/15 [29].

### 3.2. Risk of Bias

**Figure 2:**
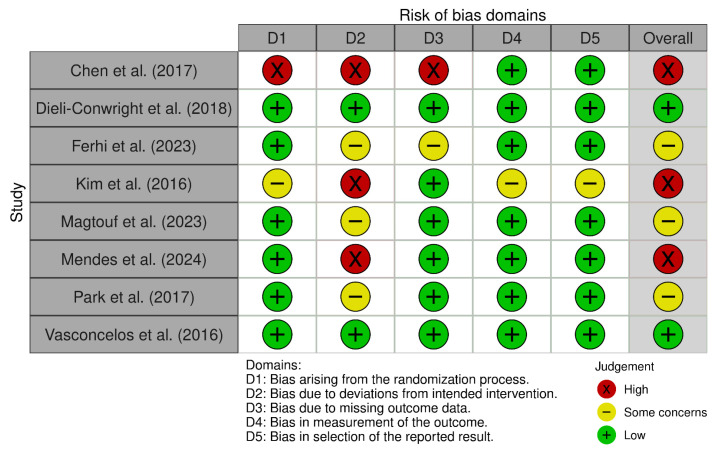
Risk of bias within studies. D1, randomization process; D2, deviations from the intended interventions; D3, missing outcome data; D4, measurement of the outcome; D5, selection of the reported results [22,23,24,25,26,27,28,29].

**Figure 3:**
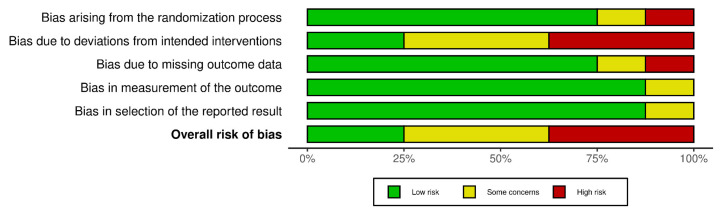
Risk-of-bias summary. Reviews the authors and judgments about each risk of bias item in each included study.

Two of the included studies were judged to have a low risk of bias [23,29]. Three studies showed some concerns [22,25,27]. Three studies were classified as having a high risk of bias [24,26,28]. The domains most frequently affected were those related to bias due to deviations from the intended intervention (D2) and bias due to missing outcome data (D3), with examples of high risk observed [22,27]. In contrast, domains concerning bias in measurement of the outcome (D4) and bias in selection of the reported result (D5) were consistently judged at low risk across most studies. Overall, with most studies showing either some concerns or high risk, the quality of evidence indicates a moderate risk of bias. Figure 2 and Figure 3 provide a detailed overview of the domain-specific judgments.

### 3.3. Studies and Sample Characteristics

All included studies were RCTs (Table 3) conducted across various geographic regions: Asia [22,25,28], Europe [24,26,27], North America [23], and South America [29]. The combined sample consisted of 453 participants, with 196 allocated to the experimental groups and 257 to the control groups. The mean participant age was 68.9 ± 11.1 years, and the majority (>80%) were female.

All interventions integrated ST with ET. The duration of the interventions ranged from 8 to 24 weeks, with training frequencies of 2 to 5 sessions per week. Session durations varied between 50 and 80 min. ST intensities ranged from 40% to 80% of one-repetition maximum (1RM) [22,23,29] or were prescribed using moderate-to-vigorous levels based on the Borg Rating of Perceived Exertion [24,26,27,28]. ET intensities ranged from 50% to 80% of the maximum heart rate [23,27]. One study did not report training intensity [25].

All studies reported adherence rates exceeding 85%. Two studies explicitly stated that no adverse events occurred during the intervention [23,25], while the remaining six studies did not provide information regarding adverse events [22,24,26,27,28,29].

### 3.4. Meta-Analysis Results

**Figure 4:**
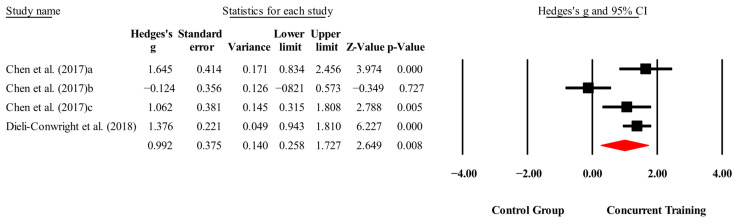
Forest plot of changes in IGF-1 in people with sarcopenic obesity participating in concurrent training compared with people with sarcopenic obesity assigned as controls. Values shown are effect sizes (Hedges’ g) with 95% confidence intervals (CIs). The size of the squares plotted reflects the statistical weight of each study [22,23].

**Figure 5:**
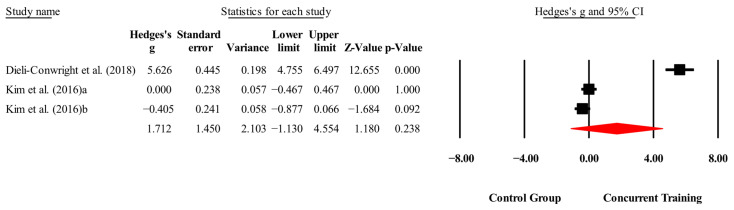
Forest plot of changes in IL-6 in people with sarcopenic obesity participating in concurrent training compared with people with sarcopenic obesity assigned as controls. Values shown are effect sizes (Hedges’ g) with 95% confidence intervals (CIs). The size of the squares plotted reflects the statistical weight of each study [23,25].

**Figure 6:**
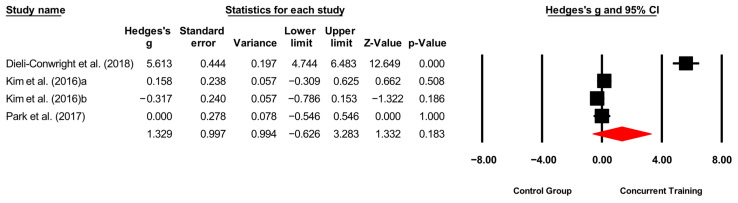
Forest plot of changes in CPR in people with sarcopenic obesity participating in concurrent training compared with people with sarcopenic obesity assigned as controls. Values shown are effect sizes (Hedges’ g) with 95% confidence intervals (CIs). The size of the squares plotted reflects the statistical weight of each study [23,25,28].

**Figure 7:**
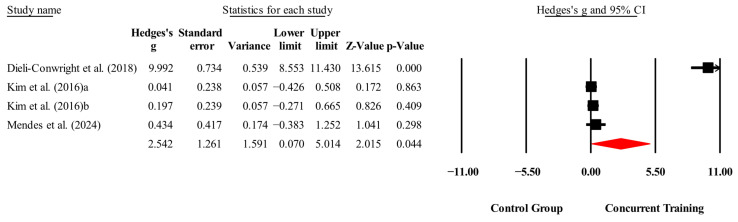
Forest plot of changes in Leptin in people with sarcopenic obesity participating in concurrent training compared with people with sarcopenic obesity assigned as controls. Values shown are effect sizes (Hedges’ g) with 95% confidence intervals (CIs). The size of the squares plotted reflects the statistical weight of each study [23,25,27].

**Figure 8:**
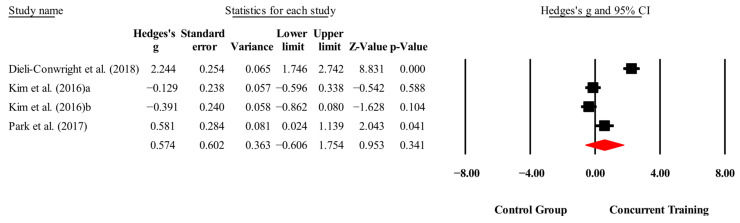
Forest plot of changes in total cholesterol in people with sarcopenic obesity participating in concurrent training compared with people with sarcopenic obesity assigned as controls. Values shown are effect sizes (Hedges’ g) with 95% confidence intervals (CIs). The size of the squares plotted reflects the statistical weight of each study [23,25,28].

**Figure 9:**
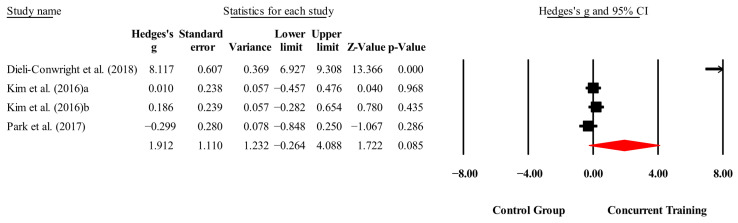
Forest plot of changes in triglycerides in people with sarcopenic obesity participating in concurrent training compared with people with sarcopenic obesity assigned as controls. Values shown are effect sizes (Hedges’ g) with 95% confidence intervals (CIs). The size of the squares plotted reflects the statistical weight of each study [23,25,28].

**Figure 10:**
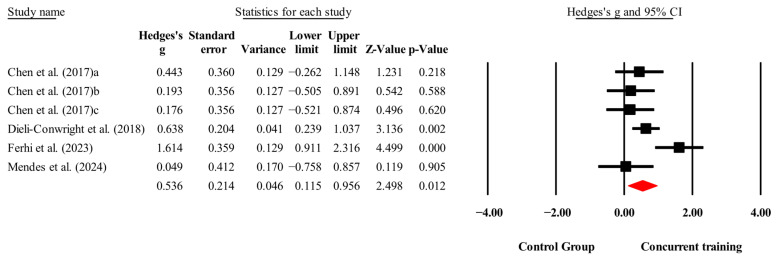
Forest plot of changes in body mass index in people with sarcopenic obesity participating in concurrent training compared with people with sarcopenic obesity assigned as controls. Values shown are effect sizes (Hedges’ g) with 95% confidence intervals (CIs). The size of the squares plotted reflects the statistical weight of each study [22,23,24,27].

**Figure 11:**
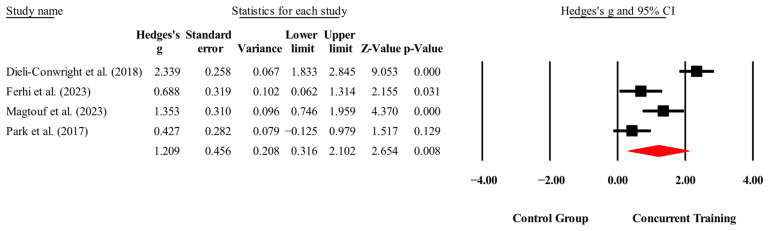
Forest plot of changes in waist circumference in people with sarcopenic obesity participating in concurrent training compared with people with sarcopenic obesity assigned as controls. Values shown are effect sizes (Hedges’ g) with 95% confidence intervals (CIs). The size of the squares plotted reflects the statistical weight of each study [23,24,26,28].

**Figure 12:**
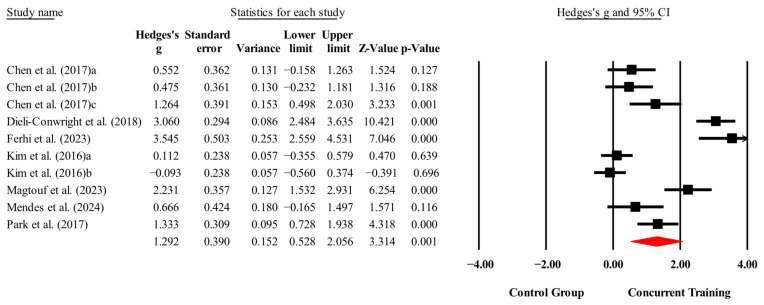
Forest plot of changes in body fat percentage in people with sarcopenic obesity participating in concurrent training compared with people with sarcopenic obesity assigned as controls. Values shown are effect sizes (Hedges’ g) with 95% confidence intervals (CIs). The size of the squares plotted reflects the statistical weight of each study [22,23,24,25,26,27,28].

**Figure 13:**
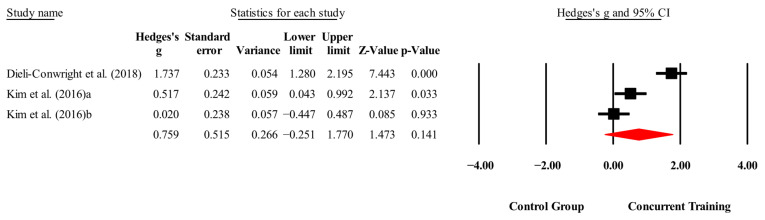
Forest plot of changes in trunk fat in people with sarcopenic obesity participating in concurrent training compared with people with sarcopenic obesity assigned as controls. Values shown are effect sizes (Hedges’ g) with 95% confidence intervals (CIs). The size of the squares plotted reflects the statistical weight of each study [23,25].

**Figure 14:**
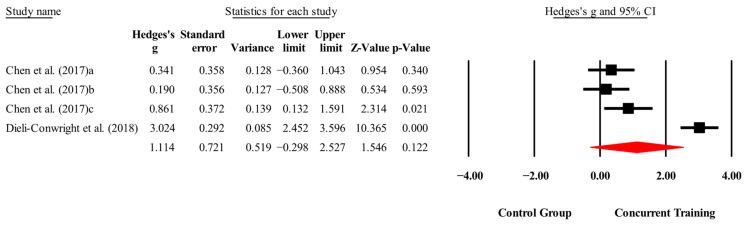
Forest plot of changes in body fat mass in people with sarcopenic obesity participating in concurrent training compared with people with sarcopenic obesity assigned as controls. Values shown are effect sizes (Hedges’ g) with 95% confidence intervals (CIs). The size of the squares plotted reflects the statistical weight of each study [22,23].

**Figure 15:**
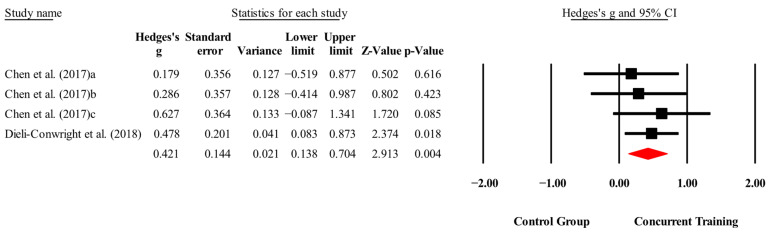
Forest plot of changes in ASM/weight in people with sarcopenic obesity participating in concurrent training compared with people with sarcopenic obesity assigned as controls. Values shown are effect sizes (Hedges’ g) with 95% confidence intervals (CIs). The size of the squares plotted reflects the statistical weight of each study [22,23].

**Figure 16:**
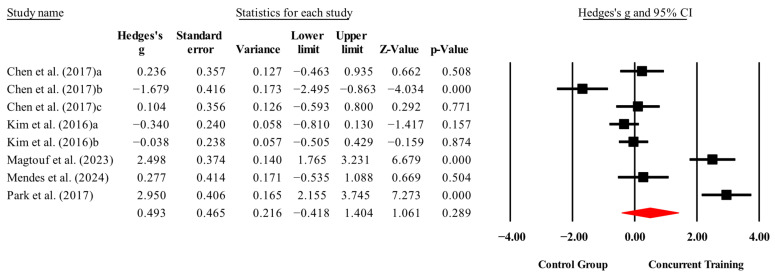
Forest plot of changes in MIHS in people with sarcopenic obesity participating in concurrent training compared with people with sarcopenic obesity assigned as controls. Values shown are effect sizes (Hedges’ g) with 95% confidence intervals (CIs). The size of the squares plotted reflects the statistical weight of each study [22,25,26,27,28].

**Figure 17:**
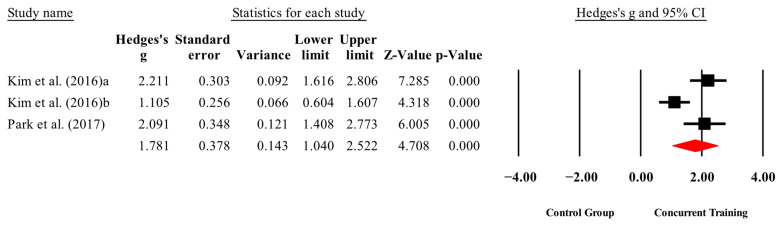
Forest plot of changes in walking speed in people with sarcopenic obesity participating in concurrent training compared with people with sarcopenic obesity assigned as controls. Values shown are effect sizes (Hedges’ g) with 95% confidence intervals (CIs). The size of the squares plotted reflects the statistical weight of each study [25,28].

**Figure 18:**
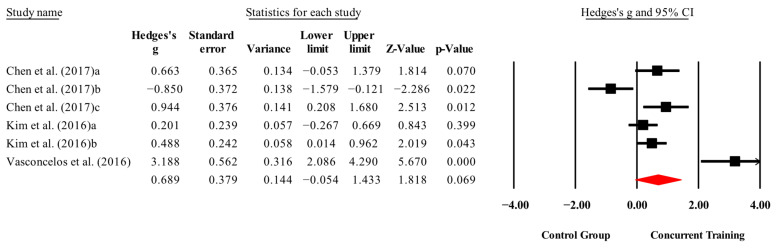
Forest plot of changes in knee extension in people with sarcopenic obesity participating in concurrent training compared with people with sarcopenic obesity assigned as controls. Values shown are effect sizes (Hedges’ g) with 95% confidence intervals (CIs). The size of the squares plotted reflects the statistical weight of each study [22,25,29].

The overall effects of CT on biomarkers, morphological variables and physical performance variables are shown in Table 4 and in Figure 4, Figure 5, Figure 6, Figure 7, Figure 8, Figure 9, Figure 10, Figure 11, Figure 12, Figure 13, Figure 14, Figure 15, Figure 16, Figure 17 and Figure 18. There were moderate to large significant effects (*p* < 0.05) in favor of CT on IGF-1, leptin, BMI, waist circumference, body fat percentage, ASM/weight, walking speed, and knee extension (ES = 0.42–2.54). However, there were no moderate to very large significant effects (*p* < 0.05) in favor of TC on IL-6, CRP, total cholesterol, triglycerides, trunk fat, body fat mass, or MIHS (ES = 0.50–1.92).

### 3.5. Sensitivity Analysis for Overall Meta-Analysis

The leave-one-out sensitivity analysis confirmed the robustness of the effects for body fat percentage and ASM/weight, as the magnitude and direction of the estimates remained stable after excluding individual studies. No significant outliers were detected in the influence diagnostics. The exclusion of influential studies affirmed the stability of significant effects for IGF-1, BMI, and waist circumference (*p* < 0.05), thereby reinforcing the reliability of these findings.

The removal of specific studies for variables including leptin, CRP, total cholesterol, triglycerides, and body fat mass resulted in a loss of statistical significance and a notable decrease in heterogeneity (I^2^ = 0%). This indicates that the observed ESs were predominantly influenced by a limited number of highly impactful studies. Sensitivity analysis was not feasible for IL-6, trunk fat, and walking speed because of the limited number of available studies, which constrained the evaluation of between-study variance.

The exclusion of two significant studies regarding knee extension strength changed both the direction and statistical significance of the effect, highlighting the instability of this outcome. These findings suggest that while some results are robust, others are significantly influenced by individual studies and warrant cautious interpretation (Table 5).

### 3.6. Meta-Analysis Subgroup

#### 3.6.1. Subgroup Analysis by Dosage Training

**Figure 19:**
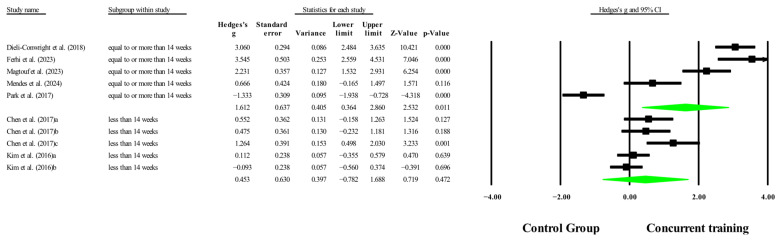
Forest plot of changes in body fat percentage in people with obesity sarcopenic participating in concurrent training compared with people with obesity sarcopenic assigned as controls with weeks training. Values shown are effect sizes (Hedges’ g) with 95% confidence intervals (CIs). The size of the squares plotted reflects the statistical weight of each study [22,23,24,25,26,27,28].

**Figure 20:**
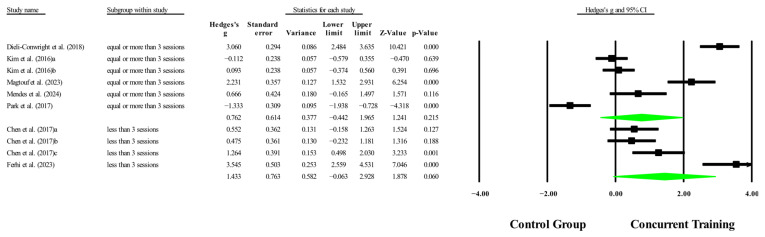
Forest plot of changes in body fat percentage in people with obesity sarcopenic participating in concurrent training compared with people with obesity sarcopenic assigned as controls with frequency training. Values shown are effect sizes (Hedges’ g) with 95% confidence intervals (CIs). The size of the squares plotted reflects the statistical weight of each study [22,23,24,25,26,27,28].

**Figure 21:**
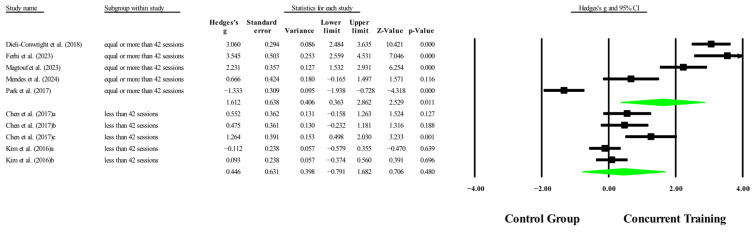
Forest plot of changes in body fat percentage in people with obesity sarcopenic participating in concurrent training compared with people with obesity sarcopenic assigned as controls with total sessions training. Values shown are effect sizes (Hedges’ g) with 95% confidence intervals (CIs). The size of the squares plotted reflects the statistical weight of each study [22,23,24,25,26,27,28].

With respect to the training dose for the variable body fat percentage, significant improvements (*p* < 0.05) were reported in favor of CT in interventions to >14 weeks, with a total number of sessions to >42 sessions with moderate to very large effects (ESs = 0.44–1.61). However, with respect to training frequency, no significant improvements (*p* > 0.05) were reported in favor of CT, with ESs ranging from large to very large (ES = 0.76–1.43), and in Figure 19, Figure 20 and Figure 21.

#### 3.6.2. Sensitivity Analysis for Subgroups

Sensitivity analyses by subgroups revealed distinct patterns of influence. In interventions lasting ≥14 weeks, the exclusion of Park, Kwon, and Park [28] reduced heterogeneity (I^2^ from 97.1% to 88.9%) and increased the ES (ES = 2.37; 95% CI: 1.24 to 3.50; *p* < 0.001). In the <14 weeks subgroup, the exclusion of Chen, Chung, Chen, Ho, and Wu [22]; and Kim, Kim, Kojima, Fujino, Hosoi, Kobayashi, Somekawa, Niki, Yamashiro, and Yoshida [25] reduced heterogeneity to 0% and rendered the effect non-significant (ES = 0.29; 95% CI: –0.05 to 0.63; *p* = 0.087). For the ≥3 sessions/week subgroup, no individual study substantially altered the ES (range: ES = 0.29 to 1.18), although Dieli-Conwright, Courneya, Demark-Wahnefried, Sami, Lee, Buchanan, Spicer, Tripathy, Bernstein, and Mortimer [23] showed notable influence without changing the direction or significance of the effect (ES = 0.29; 95% CI: –0.72 to 1.30; *p* = 0.572; I^2^ = 93.3%). In contrast, in the <3 sessions/week subgroup, the exclusion of Ferhi, Gaied Chortane, Durand, Beaune, Boyas, and Maktouf [24] reduced the effect from 1.43 to 0.74 (95% CI: 0.26 to 1.22; *p* = 0.002) and decreased heterogeneity from 89.7% to 23.2%. In the ≥ 42 sessions subgroup, exclusion of Park, Kwon, and Park [28] increased the pooled effect from 1.61 to 2.37 (95% CI: 1.24 to 3.50; *p* < 0.001), while reducing heterogeneity from 97.1% to 88.9%. In the < 42 sessions subgroup, exclusion of Chen, Chung, Chen, Ho, and Wu [22]; and Kim, Kim, Kojima, Fujino, Hosoi, Kobayashi, Somekawa, Niki, Yamashiro, and Yoshida [25] reduced the effect from 0.45 to 0.16 (95% CI: 0.13 to 0.45; *p* = 0.291). And the joint exclusion of Chen, Chung, Chen, Ho, and Wu [22]; and Kim, Kim, Kojima, Fujino, Hosoi, Kobayashi, Somekawa, Niki, Yamashiro, and Yoshida [25] further reduced heterogeneity to 0% and the effect to non-significance (ES = 0.29; 95% CI: –0.05 to 0.63; *p* = 0.098).

### 3.7. Meta-Regression

The calculation of the meta-regression was performed with at least eight studies per covariate and 10 experimental groups. Body fat percentage was considered for the meta-regression analyses, which analyzed four training variables (weeks, frequency, minutes per session, and total number of sessions; Table 6). In terms of body fat percentage during the weeks of intervention, CT was found to be a predictor of the effect of CT in the aforementioned test (*p* < 0.05).

### 3.8. Certainty of Evidence

The certainty of evidence is moderate owing to risk of bias from lack of blinding and potential protocol deviations; since no important inconsistency, indirectness, or imprecision was detected, these findings, although of small magnitude, are considered clinically relevant and underscore the need for more rigorous, larger-scale studies to confirm their true impact (Table 7).

## 4. Discussion

This meta-analysis aimed to examine the effects of CT on biomarkers (IGF-1, IL-6, CRP, leptin, total cholesterol, and triglycerides), morphological variables, and physical performance in people with sarcopenic obesity. The overall meta-analysis revealed significant effects (*p* < 0.05), ranging from moderate to large (ES = 0.42 to 2.54), in favor of CT for improving IGF-1, leptin, BMI, waist circumference, body fat percentage, the ASM/weight ratio, walking speed, and knee extension strength. However, no significant effects (*p* > 0.05) were observed for IL-6, CRP, total cholesterol, triglycerides, trunk fat, total fat mass, or MIHS (ES = 0.50 to 1.92), despite ES ranging from moderate to very large.

### 4.1. Biomarkers

#### 4.1.1. IGF-1

CT resulted in significant enhancements in circulating IGF-1 levels when compared to control conditions (*p* = 0.008; ES = 1.01). This finding corroborates earlier evidence from Annibalini et al. [30] that indicated significant increases in IGF-1 after CT in middle-aged patients with type 2 diabetes (*p* < 0.05) compared to controls.

IGF-1 serves as a key mediator in growth hormone-dependent signaling pathways, significantly contributing to cellular homeostasis and the facilitation of anabolic processes [31]. It exerts its effects by binding to the IGF-1 receptor (IGF1R) and the insulin receptor, thereby activating critical intracellular pathways, including AKT, which is vital for cell growth and proliferation [31,32]. In contrast, lower levels of IGF-1 are associated with heightened risks of sarcopenia, cardiovascular disease, and functional decline among older people [32,33,34].

This meta-analysis indicates that CT positively influences IGF-1 concentrations. Combining resistance and aerobic exercise may serve as an effective strategy to induce beneficial endocrine adaptations and potentially reduce age-related physiological decline [30]. Influence diagnostics indicated that the study by Chen, Chung, Chen, Ho, and Wu [22] significantly affected the pooled ES. Excluding this study resulted in a statistically significant ES (*p* < 0.001; ES = 1.35), with heterogeneity eliminated (I^2^ = 0%). These findings require careful interpretation, as the overall estimate may be unduly affected by a single study, which could influence the observed heterogeneity.

#### 4.1.2. IL-6

Compared with the control conditions, no significant improvements in IL-6 levels were observed in favor of CT (*p* = 0.23; ES = 1.72). Similarly, Libardi et al. [35] reported no significant changes in IL-6 after 16 weeks of CT in middle-aged males. These findings contrast with those of Trejos-Montoya [36], who conducted a systematic review comparing ET and CT in patients with coronary artery disease. That review reported significant reductions in IL-6 following CT, although no significant differences were observed compared with ET alone, and the authors suggested that these reductions might have been influenced by concomitant pharmacological treatments [36]. Elevated IL-6 concentrations are associated with frailty, reduced muscle strength, and diminished anabolic sensitivity in middle-aged and older adults [35]. While periodized training may promote anti-inflammatory effects in skeletal muscle and adipose tissue [37], few studies have specifically examined the effects of CT on systemic inflammatory biomarkers, and findings remain inconsistent [35,36].

One plausible explanation is that low-intensity or short-duration protocols may provide insufficient stimulus to lower IL-6 levels [37,38,39], whereas higher intensities and sessions lasting at least 60 min appear to enhance the physiological impact of exercise through mechanisms such as increased arterial vasodilation via nitric oxide production [40,41] and decreased secretion of vasoconstrictive neurohormones [41]. Importantly, the absence of dietary control in most interventions may also have limited the capacity to reduce systemic inflammation, since nutritional intake can directly influence IL-6 concentrations. In our meta-analysis, Dieli-Conwright, Courneya, Demark-Wahnefried, Sami, Lee, Buchanan, Spicer, Tripathy, Bernstein, and Mortimer [23] reported a significant reduction (*p* = 0.00; ES = 5.62) when a CT protocol with moderate-to-high intensities (60–80% 1RM; 65–80% HRmax) and 60 min sessions was applied, whereas Kim, Kim, Kojima, Fujino, Hosoi, Kobayashi, Somekawa, Niki, Yamashiro, and Yoshida [25] did not report training intensity, making it difficult to establish dose–response relationships.

Nevertheless, these results should be interpreted cautiously, as only two studies with a total of three experimental groups were included [23,25], and no sensitivity analysis could be performed (n = 3). Future studies should apply longer interventions with higher training intensities and structured nutritional monitoring to better determine whether CT can exert consistent anti-inflammatory effects on IL-6.

#### 4.1.3. CRP

No statistically significant reductions in CRP levels were found for CT when compared to control conditions (*p* = 0.18; ES = 1.32). This result is consistent with Libardi, De Souza, CAVAGLIERI, MADRUGA, and CHACON-MIKAHIL [35], who also reported no significant changes in CRP (*p* > 0.05) after 16 weeks of CT in middle-aged males with moderate cardiovascular risk. In contrast, Bagheri et al. [42] observed significant reductions in CRP (*p* < 0.001) after 12 weeks of CT in overweight and obese males, while Colato et al. [43] reported significant increases (*p* < 0.05) in CRP following CT in a comparable population.

Exercise-related decreases in CRP are thought to occur through inhibition of nuclear factor kappa B (NF-κB) activation and the downregulation of pro-inflammatory cytokines, including TNF-α [44,45]. However, the extent of this anti-inflammatory response is strongly influenced by training intensity and duration [35]. The absence of consistent dietary monitoring in most included trials may also have limited the ability to observe reductions in CRP, since nutritional intake can directly modulate systemic inflammation. In our meta-analysis, protocols varied considerably: Dieli-Conwright, Courneya, Demark-Wahnefried, Sami, Lee, Buchanan, Spicer, Tripathy, Bernstein, and Mortimer [23] used an 18-week program with moderate-to-high intensities (60–80% 1RM; 65–80% HRmax); Park, Kwon and Park [28] applied a 24-week intervention based on perceived exertion (RPE 13–15); and Kim, Kim, Kojima, Fujino, Hosoi, Kobayashi, Somekawa, Niki, Yamashiro, and Yoshida [25] implemented a 12-week program without reporting training intensity. Such heterogeneity in duration, intensity, and the lack of standardized nutritional control may explain the absence of consistent effects.

Additionally, acute increases in CRP can persist for several days post-exercise [35], meaning that the timing of post-intervention blood sampling could have confounded outcome measurements. Some studies observed elevated CRP levels for up to one week after training [35], yet this factor was not consistently reported or controlled. Influence diagnostics indicated that the study by Dieli-Conwright, Courneya, Demark-Wahnefried, Sami, Lee, Buchanan, Spicer, Tripathy, Bernstein, and Mortimer [23] exerted disproportionate influence on the pooled estimate; its exclusion markedly reduced heterogeneity (I^2^ = 1.56%) and rendered the overall effect nonsignificant (*p* = 0.698; ES = –0.057). Taken together, these results suggest that the lack of significant CRP reductions may be more attributable to methodological limitations—including inconsistent control of diet, variable training loads, and inadequate standardization of assessment protocols—than to a true inefficacy of CT.

#### 4.1.4. Leptin

A statistically significant decrease in leptin levels was noted for CT relative to control conditions (*p* = 0.04; ES = 2.54). This finding is consistent with previous research by Fedewa et al. [46] that indicated significant reductions in leptin levels after aerobic, resistance, and CT, with no notable differences among the exercise modalities. Leptin is crucial for body weight regulation through its modulation of triglyceride storage in non-adipose tissues that helps prevent excessive lipid accumulation [47,48]. The reductions in leptin can be partially attributed to enhancements in body composition, specifically the notable decrease in body fat percentage identified in our meta-analysis (*p* < 0.001; ES = 1.31). Furthermore, reductions in body fat, even without weight loss, have been linked to improved leptin sensitivity [46,47].

A significant methodological consideration is that the overall effect was predominantly shaped by the study conducted by Dieli-Conwright, Courneya, Demark-Wahnefried, Sami, Lee, Buchanan, Spicer, Tripathy, Bernstein, and Mortimer [23], which presented an unusually large ES (ES = 9.99). The observed changes were primarily due to a significant reduction in leptin levels in the CT group (−8.0 ng/mL) and an increase in the control group (+4.8 ng/mL), resulting in a net difference between groups of −13.4 ng/mL (95% CI = −19.7 to −9.1) with low standard deviations. The effect was statistically significant in that study, but its magnitude classified it as an outlier in influence diagnostics.

A sensitivity analysis that excluded this study resulted in an overall ES (ES = 0.16, 95% CI = −0.14 to 0.47) that was not statistically significant (*p* = 0.297), and it eliminated heterogeneity (I^2^ = 0%). The findings suggest that the observed reduction in leptin is significantly influenced by the inclusion of this particular study. Consequently, conclusions about the impact of CT on leptin levels must be approached with caution, given the instability of the pooled estimate.

#### 4.1.5. Total Cholesterol

No statistically significant improvements in total cholesterol levels were found for CT compared with control conditions (*p* = 0.34; ES = 0.57). This finding is consistent with Flores et al. [49], who reported no significant changes in young males (*p* = 0.95), and with Da Silva et al. [50], who observed no significant differences in older people. In contrast, Gálvez et al. [51] reported significant reductions in total cholesterol (*p* = 0.02) in children and adolescents with obesity following a CT program.

These discrepancies may arise from variations in participant characteristics and study designs. Total cholesterol is a nonspecific biomarker, failing to distinguish between beneficial HDL and harmful LDL fractions and, thus, limiting its sensitivity to exercise-induced alterations [52]. Moreover, dietary control varied across studies. Only Kim, Kim, Kojima, Fujino, Hosoi, Kobayashi, Somekawa, Niki, Yamashiro, and Yoshida [25] integrated nutritional supplementation with exercise, while Dieli-Conwright, Courneya, Demark-Wahnefried, Sami, Lee, Buchanan, Spicer, Tripathy, Bernstein, and Mortimer [23]; and Park, Kwon, and Park [28] did not describe structured dietary interventions. Since lipid metabolism is highly responsive to nutritional factors, this lack of standardization could have masked exercise-related improvements, particularly in populations with sarcopenic obesity [53].

The training protocols also differed in duration and intensity, which may not have reached the physiological threshold required for significant lipid changes. For example, Dieli-Conwright, Courneya, Demark-Wahnefried, Sami, Lee, Buchanan, Spicer, Tripathy, Bernstein, and Mortimer [23] implemented an 18-week intervention at moderate-to-high intensities (60–80% 1RM; 65–80% HRmax); Park, Kwon, and Park [28] used a 24-week program based on RPE (13–15); and Kim, Kim, Kojima, Fujino, Hosoi, Kobayashi, Somekawa, Niki, Yamashiro, and Yoshida [25] applied a shorter 12-week program without reporting training intensity. Such heterogeneity in program length, workload, and absence of nutritional control provides a plausible explanation for the lack of consistent results.

Influence diagnostics further indicated that the study by Dieli-Conwright, Courneya, Demark-Wahnefried, Sami, Lee, Buchanan, Spicer, Tripathy, Bernstein, and Mortimer [23] exerted disproportionate influence on the pooled estimate; exclusion reduced heterogeneity (I^2^ = 71.7%) and rendered the effect nonsignificant (*p* = 0.99; ES = 0.002). Finally, it should be acknowledged that there is considerable interindividual variability in lipid responses to exercise, which may be influenced by baseline inflammation, insulin resistance, and hepatic function. These physiological factors may blunt the lipid-lowering effects of CT interventions [54].

#### 4.1.6. Triglycerides

No significant improvements in triglyceride levels were observed in favor of CT compared with control conditions (*p* = 0.08; ES = 1.92). This result is consistent with Yu et al. [55], who also found no significant reductions (*p* = 0.07) following a combined ST + HIIT protocol in obese females with prehypertension. In contrast, Delgado-Floody et al. [56] reported significant improvements (*p* = 0.004) when CT prioritized HIIT followed by ST, compared with the reverse order (ST followed by HIIT), in females with severe and morbid obesity. Similarly, Reljic, Herrmann, Neurath, and Zopf [41] observed significant reductions in triglycerides (*p* < 0.05) in patients with metabolic syndrome, particularly when low-intensity ST was combined with HIIT compared with control conditions.

Physiologically, exercise reduces triglycerides by increasing lipoprotein lipase (LPL) activity in skeletal muscle, thereby enhancing fatty acid uptake and oxidation [57]. However, this mechanism appears to depend on reaching sufficient training volume and intensity thresholds [52,57]. In our meta-analysis, large variability in training loads was observed. Dieli-Conwright, Courneya, Demark-Wahnefried, Sami, Lee, Buchanan, Spicer, Tripathy, Bernstein, and Mortimer [23] applied an 18-week program at moderate-to-high intensities (60–80% 1RM; 65–80% HRmax); Park, Kwon, and Park [28] conducted a 24-week intervention at self-regulated intensities (RPE 13–15); and Kim, Kim, Kojima, Fujino, Hosoi, Kobayashi, Somekawa, Niki, Yamashiro, and Yoshida [25] used a shorter 12-week program without reporting training load. Such heterogeneity in program length and workload may explain the lack of consistent effects.

Additionally, triglyceride levels are strongly influenced by diet, and exercise alone may be insufficient without concomitant nutritional interventions [58,59]. The absence of standardized dietary control across studies likely contributed to the null findings. Influence diagnostics identified the study by Dieli-Conwright, Courneya, Demark-Wahnefried, Sami, Lee, Buchanan, Spicer, Tripathy, Bernstein, and Mortimer [23] as highly influential: its exclusion rendered the overall effect nonsignificant (*p* = 0.95; ES = −0.08) and eliminated heterogeneity (I^2^ = 0%). Collectively, these results suggest that the lack of significant improvements may reflect methodological limitations—including heterogeneity in training dose and the absence of dietary monitoring—rather than a true absence of exercise-induced effects. Future trials should integrate structured nutritional control with longer and more precisely prescribed CT protocols to better evaluate triglyceride responses.

This altered the magnitude, direction, or significance of the pooled effects. This highlights the limited stability of the evidence for these biomarkers and reinforces the need for caution when interpreting their results.

### 4.2. Morphological Variables

Our meta-analysis revealed significant improvements in BMI (*p* = 0.01; ES = 0.54), waist circumference (*p* = 0.008; ES = 1.22), body fat percentage (*p* = 0.001; ES = 1.31), and appendicular skeletal muscle mass relative to body weight (ASM/weight%, *p* = 0.004; ES = 0.42) in favor of CT. These findings are consistent with previous studies involving CT interventions in individuals of various ages with obesity and overweight that reported significant improvements in BMI, fat mass, lean mass, waist-to-hip ratio, and waist circumference compared with control conditions. Sarcopenic obesity has been linked to underlying metabolic dysregulation and chronic low-grade inflammation [37]. Specifically, excess adiposity leads to adipose tissue infiltration by adipocytes and macrophages, which secrete proinflammatory cytokines such as IL-6 and tumor necrosis factor-alpha (TNF-α) in response to the accumulation of free fatty acids [37,39]. This chronic inflammatory milieu negatively impacts both muscle mass and strength [3]. However, based on previous research and our current findings, CT appears to be an effective strategy for inducing significant improvements in morphological variables. From a mechanistic perspective, ET enhances mitochondrial oxidative activity [38]. It facilitates the utilization of fatty acids as energy substrates [40], a process supported by elevated circulating catecholamine levels during exercise [45]. In parallel, ST increases the basal metabolic rate, which may contribute to increased daily energy expenditure and, consequently, a gradual reduction in fat mass [40]. Collectively, these physiological mechanisms position CT as a comprehensive intervention with therapeutic potential for addressing the metabolic and functional challenges of sarcopenic obesity. On the other hand, the influence analysis for BMI identified the study by Ferhi, Gaied Chortane, Durand, Beaune, Boyas, and Maktouf [24] as influential; however, its exclusion eliminated model heterogeneity (I^2^ = 0%) and strengthened the statistical significance of the effect (*p* = 0.00; ES = 0.41). Similarly, for waist circumference, the influence analysis identified Dieli-Conwright, Courneya, Demark-Wahnefried, Sami, Lee, Buchanan, Spicer, Tripathy, Bernstein, and Mortimer [23] as an influential study. Its exclusion substantially reduced heterogeneity (I^2^ = 60.43%) while maintaining the statistical significance of the combined effect (*p* = 0.00; ES = 0.81), indicating that the overall results remained consistent without it. Regarding body fat percentage, despite the high heterogeneity observed (I^2^ = 92.9%), the sensitivity analyses confirmed the robustness of the effect, as its magnitude and direction remained stable following the exclusion of individual studies. No extreme outliers were detected in the influence diagnostics, although studies such as Dieli-Conwright, Courneya, Demark-Wahnefried, Sami, Lee, Buchanan, Spicer, Tripathy, Bernstein, and Mortimer [23]; and Ferhi, Gaied Chortane, Durand, Beaune, Boyas, and Maktouf [24] exhibited greater statistical weight. Finally, for ASM/weight, the sensitivity analysis showed that removing any single study did not alter the significance of the overall effect, further supporting the robustness of this finding. The influence diagnostics also indicated a proportional contribution of each study to the pooled estimate, with no single study disproportionately driving the effect. Taken together, the sensitivity and influence analyses confirm the robustness of our findings and support the positive impact of CT on BMI, waist circumference, body fat percentage, and ASM relative to body weight.

In contrast, our meta-analysis did not reveal significant effects on body fat mass (*p* = 0.12; ES = 1.13) or trunk fat (*p* = 0.14; ES = 0.76). This may be attributed to the need for longer intervention durations (>18 weeks) to induce detectable changes in specific fat compartments [44,60]. For example, while variables such as waist circumference or body fat percentage reflect global or indirect changes in adiposity, trunk fat, and absolute fat mass represent more specific and stable measures that are less sensitive to short-term variations [61]. Additionally, the magnitude of change in these compartments may be influenced by uncontrolled factors, such as caloric intake and adherence to the intervention protocol, in the included studies. Finally, the precision of the assessment tools used to evaluate these compartments may have impacted the ability to detect changes, particularly in cases where indirect or less sensitive methods, such as bioelectrical impedance analysis, were employed [62]. Taken together, these factors may help explain the lack of significant findings for these variables. Regarding the influence analysis for body fat mass, the study by Dieli-Conwright, Courneya, Demark-Wahnefried, Sami, Lee, Buchanan, Spicer, Tripathy, Bernstein, and Mortimer [23] was identified as highly influential. Its exclusion significantly altered the overall effect estimate (*p* = 0.03; ES = 0.45), suggesting that this study contributed disproportionately to the observed ES. Moreover, heterogeneity was eliminated (I^2^ = 0%) after its removal. These findings indicate that the results for body fat mass are heavily dependent on this particular study, limiting the robustness and generalizability of the conclusions. In contrast, for the trunk fat variable, sensitivity and influence analyses could not be conducted due to the limited number of studies included in the meta-analysis (n = 3). Excluding a single study would leave only two observations, severely restricting the model’s capacity to accurately estimate heterogeneity and assess the stability of the pooled effect. Therefore, results should be interpreted with caution, taking this methodological limitation into account.

### 4.3. Physical Performance

Compared with control conditions, CT conditions significantly improved walking speed (*p* = 0.00; ES = 1.80) and knee extension strength (*p* = 0.02; ES = 0.76). These findings are consistent with those reported by Khalafi, Sakhaei, Rosenkranz, and Symonds [8] in a systematic review and meta-analysis, which demonstrated significant improvements in lower-limb muscle strength following CT (*p* = 0.001) compared with ET alone in middle-aged and older people. CT was also found to be as effective as ST for improving lower-limb muscle strength. Similarly, Markov et al. [63] reported significant gains in maximal isokinetic torque, isometric strength, and 1RM of knee extensors following CT (*p* < 0.01) compared with control groups in adults of similar age ranges. Our findings may be explained by the complementary physiological adaptations induced by CT. On the one hand, ST promotes increases in muscle protein synthesis, cross-sectional area (CSA), neuronal excitability, and rate of force development (RFD), which translate into increased muscle strength [64,65]. On the other hand, ET stimulates mitochondrial biogenesis, angiogenesis, and cardiovascular efficiency, thereby increasing the oxidative capacity of skeletal muscle [52,66]. In this context, the combination of both stimuli within CT enables the simultaneous development of these adaptations, supporting improvements in both strength-related parameters and locomotor performance in older people with sarcopenic obesity [53]. The influence analysis for knee extension strength revealed that the studies by Chen, Chung, Chen, Ho, and Wu [22]; and Vasconcelos, Dias, Araújo, Pinheiro, Moreira, and Dias [29] had a disproportionate impact on the meta-analysis results. The simultaneous exclusion of both studies improved the statistical significance of the model (*p* = 0.001; ES = 0.48) and substantially reduced heterogeneity (I^2^ = 4.39%). These findings suggest that the presence of these studies may have distorted the overall effect estimate, compromising the stability and generalizability of the observed outcome. In contrast, due to the limited number of studies included in the walking speed meta-analysis (n = 3), sensitivity and influence analyses could not be conducted. As previously discussed, excluding even a single study would leave only two data points, severely limiting the model’s ability to accurately estimate heterogeneity and assess the stability of the overall effect.

On the other hand, no significant improvements were identified in MIHS following CT (*p* = 0.29; ES = 0.50) compared with the control conditions. This contrasts with the findings of Khalafi, Sakhaei, Rosenkranz, and Symonds [8], who reported significant increases in upper-limb muscle strength in favor of CT (*p* = 0.001) compared with ET alone in middle-aged and older people. CT was also shown to be as effective as ST in improving upper-limb muscle strength. MIHS has been reported to gradually decline with age, beginning as early as the third decade of life and accelerating after the age of 50 [54]. However, ST has demonstrated the potential to mitigate and partially reverse this decline in muscle strength associated with aging [67,68]. Despite this, our meta-analysis did not reveal significant improvements in MIHS in favor of CT, which may be attributed to the lack of stimulus specificity in the interventions analyzed [22,25,26,27,28]. Increases in MIHS are thought to arise primarily from repeated muscular stimulation through exercises that consistently involve gripping and pulling actions [57,69]. While MIHS is recognized as an important health marker in older people and individuals with sarcopenic obesity [58,59], lower-limb muscle strength may be even more relevant in this population [8], as it has a greater impact on fall prevention and plays a critical role in maintaining autonomy and independence in activities of daily living [70]. In this context, our findings suggest that CT does not significantly enhance MIHS and may require more targeted training strategies to elicit meaningful gains, particularly when aiming to improve both general health indicators and functional capacity across body segments. The influence analysis for MIHS identified Park, Kwon, and Park [28] as having a substantial impact on the overall effect estimate. However, its exclusion did not alter the statistical significance of the model (*p* = 0.70; ES = 0.15), although it slightly reduced heterogeneity (I^2^ = 90.5%). These findings suggest that while this study broadly defines the magnitude of the effect, the overall meta-analysis results are not critically dependent on its inclusion.

### 4.4. Meta-Analysis Subgroup

#### 4.4.1. Subgroup Analysis by Dosage Training

Concerning the training dose for the outcome of body fat percentage, significant improvements were observed in favor of CT (*p* < 0.05) for interventions lasting more than 14 weeks and comprising more than 42 total sessions, with moderate to very large ESs (ES = 0.44–1.61). These findings are consistent with those of Khalafi, Sakhaei, Rosenkranz, and Symonds [8], who, in a meta-analysis on the effects of CT in middle-aged and older people, reported significant improvements in body fat percentage (*p* = 0.001) in subgroup analyses for long-term interventions (≥24 weeks) compared with ET alone. Similarly, Khalafi, Sakhaei, Rosenkranz, and Symonds [8] reported significantly greater reductions in fat mass (*p* = 0.01) following midterm interventions (<24 weeks) than aerobic training alone. Our findings align with previous evidence indicating that training duration and cumulative volume are critical factors for eliciting favorable changes in body composition, particularly in adults with sarcopenic obesity [71], a population characterized by greater resistance to metabolic changes and attenuated anabolic responses [71]. The influence analysis revealed that in the ≥14-week subgroup, the study by Park, Kwon, and Park [28] had a disproportionate impact on the overall effect estimate and heterogeneity. Its exclusion reduced model heterogeneity (I^2^ = 88.94%) and increased the precision of the effect (*p* < 0.001; ES = 2.37). Similarly, in the subgroup with ≥42 total sessions, Park, Kwon, and Park [28] were again identified as having a considerable influence on the meta-analysis estimate. Removing this study increased both the magnitude and precision of the effect (*p* < 0.001; ES = 2.37), while reducing heterogeneity (I^2^ = 88.9%). Although the combined effects for both subgroups remained positive and statistically significant, these results should be interpreted with caution due to the model’s sensitivity to this particular study.

However, regarding training frequency, no significant improvements (*p* > 0.05) were observed in favor of CT compared with the control conditions, despite a large to very large ES (ES = 0.76–1.43). This discrepancy may be attributed to the interaction between frequency, total training volume, and adherence, as well as methodological heterogeneity across studies. In this context, training frequency alone may not ensure cumulative physiological benefits unless it is accompanied by sufficient overall volume and well-structured intensity and progression [60,71]. Taken together, these findings underscore the importance of the total intervention duration and cumulative workload as key components for achieving meaningful improvements in morphological variables through CT in older people with sarcopenic obesity. The influence analysis for training frequency subgroups revealed divergent outcomes. In the subgroup of ≥3 sessions/week, the study by Dieli-Conwright, Courneya, Demark-Wahnefried, Sami, Lee, Buchanan, Spicer, Tripathy, Bernstein, and Mortimer [23] exerted a disproportionate influence on the pooled effect. However, its exclusion did not reverse the direction of the impact or substantially reduce its magnitude (*p* = 0.57; ES = 0.29), although heterogeneity remained high (I^2^ = 93.3%). Therefore, the overall result for this subgroup can be considered robust, yet it warrants cautious interpretation due to the significant weight of this specific study. In contrast, for the <3 sessions/week subgroup, a sensitivity analysis, showed that removing the study by Ferhi, Gaied Chortane, Durand, Beaune, Boyas, and Maktouf [24] had a substantial impact on the results. Upon its exclusion, the pooled estimate became statistically significant (*p* = 0.002; ES = 0.74), with a marked reduction in heterogeneity (I^2^ = 23.2%). Influence diagnostics confirmed this study as highly influential. While the original model for this subgroup only approached significance (*p* = 0.06; ES = 1.43), the sensitivity analysis shifted the result into the significant range. Consequently, these findings should be interpreted with caution, as the observed effects rely heavily on a single influential study.

#### 4.4.2. Meta-Regression

The meta-regression analysis identified intervention duration as a significant predictor of improvements in body fat percentage (*p* < 0.05), accounting for 32% of the observed variance. As previously discussed, this finding suggests that prolonged interventions (>14 weeks) are associated with greater reductions in body fat among adults with sarcopenic obesity. From a physiological standpoint, extending the duration of training may increase the activation of pathways involved in fatty acid oxidation, such as PGC-1α and AMPK, which promote mitochondrial biogenesis, metabolic efficiency, and the lipolytic capacity of skeletal muscle [72]. Additionally, sustained exercise contributes to improved insulin sensitivity, the regulation of hormones such as leptin, and reductions in chronic low-grade inflammation, which are key modulators of lipid metabolism in people with sarcopenic obesity [73]. In this context, longer CT interventions may not only increase cumulative energy expenditure but also foster endocrine and molecular adaptations that facilitate adipose tissue mobilization and reduction [60,71].

In summary, previous meta-analyses examining exercise interventions in sarcopenic obesity have generally reported beneficial effects of ST, ET, or CT on body composition and physical performance, but they have provided limited exploration of training dosage parameters. Our work complements these reviews by conducting subgroup analyses and a meta-regression focused on intervention frequency, duration, and total number of sessions. Notably, we found that interventions lasting more than 14 weeks and including ≥42 sessions were associated with more consistent reductions in body fat percentage, suggesting a dose–response effect of CT. However, insufficient reporting of training intensity across several trials prevented a robust analysis of this parameter, which remains a critical gap for future research. In this way, the present meta-analysis not only corroborates previous findings but also extends them by clarifying the role of training duration and volume, while highlighting the need for more standardized reporting of exercise intensity.

Regarding the certainty of evidence in this meta-analysis, it was reported as moderate, which allows recommendations to be made on the use of CT in biomarkers, morphological variables, and physical performance in people with sarcopenic obesity with a total of 453 participants, but with some caution. Similar results to those reported by da Silva Gonçalves, Santos Lopes da Silva, Rodrigues Benjamim, Tasinafo Junior, Bohn, Ferreira Abud, Ortiz, and de Freitas [9] in people with sarcopenic obesity with intervention CT showed moderate certainty of evidence in the variables of body fat percentage and fat-free mass with a total of 978 participants. However, in a meta-analysis conducted by Tian, Li, Zhang, Liu, Huang, Yu, Wu, Cao, Peng, and García-Ramos [10] in people with sarcopenic obesity, with a total of 1457 participants comparing different non-pharmacological therapies (physical exercise and nutrition), low-to-moderate certainty of evidence was reported in anthropometric, physical, and physiological variables.

### 4.5. Limitations and Strengths

Our meta-analysis presents the following limitations: (i) moderate risk of bias, with most studies presenting some concerns and only a few classified as high risk; (ii) high heterogeneity across several variables in the overall meta-analysis; (iii) the inability to conduct subgroup analyses for some biomarkers and physical performance outcomes; (iv) high sensitivity of several outcomes and subgroups to individual studies, as revealed by leave-one-out and influence diagnostics, which identified disproportionately influential studies whose exclusion altered the magnitude, direction, or heterogeneity of the pooled effects; (v) missing data regarding training intensity in some trials, which may have influenced the analyses and limited our ability to examine this factor as a moderator; and (vi) most participants were older women (≥80%), thus restricting the generalizability of our findings. Thus, caution is needed when extrapolating the observed effects of CT to men or to younger adults, as sex- and age-related physiological differences may influence adaptive responses. Conversely, the strengths of this meta-analysis include (i) the use of six generic scientific databases, namely PubMed, Medline, CINAHL Complete, Scopus, the Cochrane Library, and Web of Science (Core Collection); (ii) a methodological quality exceeding 60% across the included studies; (iii) adherence to rigorous methodological standards, including PRISMA, PROSPERO, TESTEX, RoB 2, and GRADE; (iv) the implementation of subgroup meta-analysis based on training duration; (v) the execution of meta-regression analyses for body fat percentage, incorporating four training-related covariates (weeks, frequency, session duration, and total number of sessions); and (vi) the application of leave-one-out sensitivity and influence analyses to assess the robustness of results and identify studies with disproportionate impact on pooled estimates, thereby enhancing the transparency and interpretability of the findings.

### 4.6. Practical Applications

CT emerges as a highly effective non-pharmacological intervention for people with sarcopenic obesity, offering significant applications across clinical, exercise, and public health domains. In clinical management and therapeutic prescription, CT should be considered a cornerstone strategy due to its ability to improve morphological variables, specifically reducing BMI, waist circumference, and overall body fat. It also enhances appendicular skeletal muscle mass, walking speed, and knee extension strength, which are vital for mobility and fall prevention in this population. Furthermore, CT has a positive influence on key biomarkers, such as IGF-1 and leptin, providing measurable physiological benefits. However, clinicians should note that CT alone may not significantly alter IL-6, C-reactive protein, total cholesterol, triglycerides, trunk fat, body fat mass, or MIHS, suggesting the need for complementary interventions or targeted exercise to address these outcomes.

For exercise professionals and program design, our findings underscore the importance of intervention duration. Optimal reductions in body fat are achieved with longer CT protocols, typically exceeding 14 weeks and including more than 42 total sessions. Programs should consistently combine strength and endurance components, comprising 2–5 weekly sessions, each lasting 50–80 min, with intensities tailored to individual capabilities (e.g., 40–80% of 1RM for strength and 50–80% of HRmax for endurance). Where improving MIHS is a specific goal, targeted exercises beyond the general CT regimen are necessary, as general CT may not yield significant gains in this area.

From a public health perspective, the robust evidence supporting CT warrants its integration into national and local physical activity guidelines for people with sarcopenic obesity. Importantly, pragmatic adaptations such as home-based programs using elastic bands, bodyweight exercises, or walking combined with simple aerobic activities could improve feasibility and adherence, particularly for individuals with limited access to supervised facilities. Developing accessible community- and home-based CT strategies would promote health and functional independence in this population. Policymakers could also explore healthcare reimbursement mechanisms for supervised or partially supervised CT, making this intervention more widely available and affordable.

In addition, future research should prioritize longer interventions exceeding 24 weeks, with strict nutritional control and standardized reporting of training intensity. Studies should also aim to include a more balanced representation of men and younger adults to improve generalizability, and they should incorporate more comprehensive biomarker characterization to better elucidate the mechanistic pathways underlying the effects of CT.

## 5. Conclusions

CT leads to clinically significant improvements in biomarkers such as IGF-1 and leptin, which are strongly associated with favorable changes in morphological variables, including increased muscle mass and reduced body fat percentage, as well as significant improvements in lower-body muscle strength and mobility. These benefits have meaningful implications for healthcare, given that obesity and sarcopenia are major global challenges. With regard to training dosage, CT interventions lasting at least 14 weeks with ≥42 sessions appear to show more consistent effects on body fat reduction in people with sarcopenic obesity. However, the high heterogeneity observed in several outcomes (I^2^ often >90%) substantially limits the robustness of these findings. Therefore, while CT shows promising effects, the conclusions should be interpreted with caution, and further well-designed, long-term trials are required to confirm and strengthen the evidence base.

## Figures and Tables

**Table 1:** Selection criteria used in the systematic review.

Category	Inclusion Criteria	Exclusion Criteria
Population	Persons to have a combination of obesity, sarcopenia, and no other diseases, such as fractures and heart failure. Participants were required to be adults (age ≥ 18 years), but there were no restrictions by sex or setting (such as hospitals, communities, or nursing homes).	Population under 18 years of age only with obesity and/or sarcopenia. Persons ≥18 years of age apparently healthy.
Intervention	Interventions included strength training (resistance training, elastic band training, and progressive resistance training) and endurance training (aerobic training, treadmill training, walking training, gait training), with a minimum of 4 weeks of intervention at a frequency of 1 session per week with a minimum of 30 min per session.	Interventions that do not use strength training and endurance training. There are no details of the intervention procedure.
Comparator	Interventions with or without an active/inactive control groups or placebo.	Observational studies (i.e., cross-sectional, retrospective, and prospective studies) that do not include structured comparison pre/post analysis.
Outcome	Primary outcomes: biomarkers (e.g., IGF-1, IL-6, leptin, C-reactive protein, cholesterol, glycemia, and triglycerides), morphological variables (e.g., body mass index, waist circumference, body fat percentage, skeletal muscle mass index, and bone mineral density); physical performance (e.g., MIHS, gait speed, and knee extension). To determine the diagnosis of sarcopenic obesity, they followed the guidelines of the European Sarcopenia Group and/or Asian Sarcopenia Group and/or Brazilian Sarcopenia Group.	Lack of baseline data and/or follow-ups.
Study design	Experimental design studies (randomized controlled trials) with pre- and post-assessments.	Non-randomized controlled trials, cross-sectional, retrospective, and prospective studies.

IGF-1: insulin-like growth factor-1; IL-6: interleukin-6; MIHS: maximal isometric handgrip strength.

**Table 2:** Study quality assessment according to the TESTEX scale.

Eligibility Criteria Specified	Randomly Allocated Participants	Allocation Concealed	Groups Similar at Baseline	Assessors Blinded	Outcome Measures Assessed >85% of Participants *	Intention to Treat Analysis	Reporting of Between Group Statistical Comparisons	Point Measures and Measures of Variability Reported **	Activity Monitoring in Control Group	Relative Exercise Intensity Reviewed	Exercise Volume and Energy Expended	Overall TESTEX #
Chen, Chung, Chen, Ho, and Wu [22]	Yes	Yes	Yes	Yes	Yes (1)	No	Yes	Yes (2)	Yes	Yes	Yes	12/15
Dieli-Conwright, Courneya, Demark-Wahnefried, Sami, Lee, Buchanan, Spicer, Tripathy, Bernstein, and Mortimer [23]	Yes	Yes	Yes	No	Yes (2)	No	Yes	Yes (2)	Yes	Yes	Yes	12/15
Ferhi, Gaied Chortane, Durand, Beaune, Boyas, and Maktouf [24]	Yes	Yes	Yes	Yes	Yes (1)	No	Yes	Yes (2)	Yes	Yes	Yes	12/15
Kim, Kim, Kojima, Fujino, Hosoi, Kobayashi, Somekawa, Niki, Yamashiro, and Yoshida [25]	Yes	Yes	Yes	No	Yes (2)	No	Yes	Yes (2)	Yes	No	Yes	11/15
Magtouf, Chortane, Chortane, Boyas, Beaune, Durand, and Maktouf [26]	Yes	Yes	Yes	Yes	Yes (1)	No	Yes	Yes (2)	Yes	Yes	Yes	12/15
Mendes, Carvalho, Bravo, Martins, and Raimundo [27]	Yes	Yes	Yes	Yes	Yes (1)	No	Yes	Yes (2)	Yes	Yes	Yes	12/15
Park, Kwon, and Park [28]	Yes	Yes	Yes	Yes	Yes (1)	No	Yes	Yes (2)	Yes	Yes	Yes	12/15
Vasconcelos, Dias, Araújo, Pinheiro, Moreira, and Dias [29]	Yes	Yes	Yes	Yes	Yes (1)	Yes	Yes	Yes (2)	Yes	Yes	Yes	13/15

* Three points are possible: one point if adherence was 85%, one point if adverse events were reported, and one point if exercise attendance was reported. ** Two points possible: one point if the primary outcome is reported and one point if all other outcomes are reported. # Total out of 15 points. TESTEX: Tool for assessing study quality and reporting in exercise.

**Table 3:** Effects of concurrent training on body composition, physical performance, and biomarkers in people with sarcopenic obesity.

Autor	Country	Study Design	Sex (Age)	Type of Training (Sample Size)	Diagnostic Criteria for Obesity Sarcopenic	Morphological Assessment Tool	Training			Intensity	Morphological Variables	Physical Performance	Biomarkers	Adverse Events and Adherence
							Weeks	Frequency	Minutes					
Chen, Chung, Chen, Ho, and Wu [22]	Taiwan	RCT	Women 80% Men 20% CT: 68.5 ± 2.7 ST: 70.3 ± 11.2 AT: 62.8 ± 9.4 CG: 69.2 ± 9.6	CT (15): strength training combination aerobic training ST (15): strength training with free weights AT (15): combination of dance steps and jumping exercises CG (15): continued with their daily life activities	Sarcopenia is defined as appendicular skeletal muscle mass (ASM) (kg)/weight (kg) 9100%. The determining threshold value is ≤32.5% for men and ≤25.7% for women. Obesity indicators are body mass index (BMI) ≥ 25 kg/m^2^,13 and visceral fat area (VFA) ≥ 100 cm^2^.	Electrical bioimpedance (Inbody 720, Biospace Inc., Cerritos, CA, USA)	8	2	60	60–70% 1RM Endurance training moderate intensity	Weight (kg) SMM (kg) ASM/weight (%) BFM (kg) BMI (kg/m^2^) BF (%) VFA (cm^2^)	Back extensor (kg) Knee extensor (kg) MIHS (kg)	IGF-1 (ng/mL)	NR >85
Dieli-Conwright, Courneya, Demark-Wahnefried, Sami, Lee, Buchanan, Spicer, Tripathy, Bernstein, and Mortimer [23]	United States	RCT	Women 100% CT: 52.8 ± 10.6 CG: 53.6 ± 10.1	CT (50): strength training with free weights and endurance training in cycling CG (50): continued with their daily life activities	Sarcopenic obesity was defined as appendicular skeletal mass index, <5.45 kg/m^2^ and BMI ≥ 30.0 kg/m^2^.	DEXA (Lunar GE iDXA; Fairfield, CT, USA)	18	3	60	60–80% 1RM 65–80% heart rate maximum	Waist circumference (cm) BMI (kg/m^2^) ASM/weight (%) BFM (kg) Lean mass (kg) BF (%) Trunk fat (kg)	NR	HDL-C (mg/dL) Total cholesterol (mg/dL) Triglycerides (mg/dL) Glucose (mg/dL) ATP III-score IGF-1 (ng/mL) IGFBP-3, (ng/mL) CRP (mg/L) Leptin (ng/mL) Adiponectin (mg/mg) IL-6 (pg/mL) IL-8 (pg/mL) TNF-a (pg/mg) SHBG (nmol/L) Estradiol (pg/mL) Free testosterone (pg/mL)	No adverse events occurred >85
Ferhi, Gaied Chortane, Durand, Beaune, Boyas, and Maktouf [24]	France	RCT	NR CT: 74.1 ± 3.7 CG: 76.6 ± 5.6	CT (20): strength training with free weights and walking exercises CG (20): continued with their daily life activities	Sarcopenic obesity was defined having a BMI > 30 kg/m^2^, a Handgrip force (HF) < 17 N, gait speed < 1.0 m/s, being	Electrical bioimpedance (Tanita; SC 240-Class III; Tanita Europe B.V., Amsterdam, The Netherlands).	24	2	60	6 (RPE of the 10 points)	Weight (kg) BMI (kg/m^2^) BF (%) Waist circumference (cm)	Knee extensor (kg) Absolute PT 60 s 1 (Nm) Relative PT 60 s 1 (Nm/kg 100)	NR	NR >85
Kim, Kim, Kojima, Fujino, Hosoi, Kobayashi, Somekawa, Niki, Yamashiro, and Yoshida [25]	Japan	RCT	Women 100% CT: 81.4 ± 4.3 N: 81.2 ± 4.9 CG: 81.1 ± 5.1	CT (35): strength training with elastic band training and endurance training in cycling N (34): Supplementation with amino acids and tea Catechin CG (34): A general health education class	Sarcopenic obesity was operationally defined as body fat percent of 32% or greater, combined with skeletal muscle mass index less than 5.67 kg/m^2^; body fat percent of 32% or greater and grip strength less than 17.0 kg; and body fat percent of 32% or greater and walking speed under 1.0 m/s.	Electrical bioimpedance (Inbody 720, Biospace, Seoul, Republic of Korea)	12	3	60	NR	ASM (kg) BF (%) Trunk fat (kg) Total body fat (kg) Weight (kg)	Walking speed (m/s) MIHS (kg) Knee extension (kg)	Total cholesterol (mg/dL) Triglycerides (mg/dL) Hemoglobin A1c (%) Leptin (ng/mL) Cystatin C (mg/L) Vitamin D (ng/mL) IL-6 (pg/mL) CRP (mg/L)	No adverse events occurred >85
Magtouf, Chortane, Chortane, Boyas, Beaune, Durand, and Maktouf [26]	France	RCT	NR CT: 76.3 ± 3.5 CG: 75.9 ± 5.4	CT (25): strength training with free weights with elastic band training and walking exercises CG (25): continued with their daily life activities	Sarcopenic obesity was defined having a BMI > 30 kg/m^2^, a Handgrip force (HF) < 17 N, gait speed < 1.0 m/s, being	Electrical bioimpedance (Tanita; SC 24, Amsterdam, The Netherlands)	16	3	60	6 (RPE of the 10 points)	Body mass (kg) Waist circumference (cm) Body mass (kg) BF (%) FBM (kg) LBM (kg)	TUG (s) Gait speed (m/s) MIHS (kg) Romberg test (s)	NR	NR >85
Mendes, Carvalho, Bravo, Martins, and Raimundo [27]	Portugal	RCT	Women 85% Men 15% CT: 44.08 ± 13.2 CG: 50.4 ± 11.1	CT (12): strength training with free weights with machine and endurance training in cycling CG (10): continued with their daily life activities	Sarcopenic obesity was defined as a high BMI or waist circumference, combined with low muscle mass and low muscle strength	DEXA (DXA, Hologic QDR, Hologic, Inc., Bedford, MA, USA)	16	3	55	4–6 (RPE of the 10 points) 50–60% heart rate maximum	BMI (kg/m^2^) Weight (kg) BF (%) Lean mass (kg) BMC (kg) BMD (g/cm^2^)	MIHS (kg)	Leptin (ng/mL) Ghrelin (pg/mL)	NR >85
Park, Kwon, and Park [28]	South Korea	RCT	Women 100% CT: 73.5 ± 7.1 CG: 74.7 ± 5.1	CT (25): strength training with elastic band training and walking exercises CG (25): continued with their daily life activities	Sarcopenic obesity was defined as a body mass index (BMI) ≥ 25.0 kg/m^2^ and ASM/weight < 25.1%	Electrical bioimpedance (Inbody 720, Biospace, Seoul, Republic of Korea)	24	5	50 to 80	13–15 (RPE of the 20 points)	Waist circumference (cm) BF (%) ASM (kg)	MIHS (kg) Sit and Reach (cm) Walking speed (m/s) 2-Minute step	HDL-C (mg/dL) LDL-C (mg/dL) Total cholesterol (mg/dL) Triglycerides (mg/dL) CRP (mg/L)	NR >85
Vasconcelos, Dias, Araújo, Pinheiro, Moreira, and Dias [29]	Brazil	RCT	Women 100% CT: 72 ± 4.6 CG: 72 ± 3.6	CT (14): strength training with free weights and walking exercises CG (14): continued with their daily life activities	Sarcopenic obesity was defined by a body mass index (BMI) ≥30 kg/m^2^ and handgrip strength ≤21 kg	Calibrated balance (FilizolaTM, São Paulo, SP, Brazil)	10	2	60	40–75% 1RM	NR	Knee extension (kg) Gait speed (m/s)	NR	NR >85

ASM: appendicular skeletal muscle mass; PT: Peak Torque; BFM: body fat mass; SMM: skeletal muscle mass; VFA: visceral fat area; ALM: appendicular lean mass; TUG  =  timed up-and-go test; FBM: fat body mass; LBM: lean body mass; BMD: bone mineral density; BMC: bone mineral content; CRP: C-reactive protein.

**Table 4:** Effects of concurrent training on biomarkers, morphological variables, and physical performance in people with sarcopenic obesity.

Biomarkers							
	n ^a^	Model Effect	ES (95%CI)	*p*	I^2^ (%)	Egger’s Test (*p*)	RW (%)
IGF-1(ng/mL)	2,2,4, 160.	Random	1.01 (0.26 to 1.75)	**0.008**	79.8	0.002	43.4 to 62.4
IL-6 (pg/mL)	2,2,3, 203.	Random	1.72 (−1.14 to 4.59)	0.23	98.6	0.000	0.81 to 4.97
CRP (mg/L)	3,3,4, 288.	Random	1.32 (−0.63 to 3.31)	0.18	97.9	0.000	1.31 to 7.70
Leptin (ng/mL)	3,3,4, 260.	Random	2.54 (0.07 to 5.01)	**0.04**	98.2	0.000	1.59 to 14.9
Total cholesterol (mg/dL)	3,3,4, 288.	Random	0.57 (−0.60 to 1.75)	0.34	95.5	0.000	1.58 to 3.81
Triglycerides (mg/dL)	3,3,4, 288.	Random	1.92 (−0.27 to 4.12)	0.08	98.2	0.000	21.4 to 80.3
**Morphological variables**							
BMI (kg/m^2^)	4,4,6, 252.	Random	0.54 (0.12 to 0.97)	**0.01**	59.9	0.02	32.1 to 34
Waist circumference (cm)	4,4,4, 240.	Random	1.22 (0.32 to 2.12)	**0.008**	89.6	0.000	5.80 to 9.65
Body fat (%)	7,7,10, 460.	Random	1.31 (0.53 to 2.09)	**0.001**	92.9	0.000	8.36 to 20
ASM/weight (%)	2,2,4, 160.	Fixed	0.42 (0.14 to 0.71)	**0.004**	**0.00**	0.80	9.49 to 19.8
Trunk fat (kg)	2,2,3, 203.	Random	0.76 (−0.25 to 1.78)	0.14	92.8	0.000	2.38 to 4.13
Body fat mass (kg)	2,2,4, 160.	Random	1.13 (−0.29 to 2.56)	0.12	94.2	0.000	2.12 to 4.88
**Physical performance**							
Walking speed (m/s)	2,2,3, 153.	Random	1.80 (1.05 to 2.55)	**0.000**	79	0.008	12.2 to 23.8
MIHS (kg)	5,5,8, 350.	Random	0.50 (−0.42 to 1.43)	0.29	93.5	0.000	2.22 to 10.2
Knee extension (kg)	3,3,6, 231.	Random	0.76 (0.09 to 1.42)	**0.02**	85.8	0.000	6.57 to 15.3

n ^a^: number of studies, number of experimental groups, number of control groups, number of participants; ASM: appendicular skeletal muscle mass; IGF-1: insulin-like growth factor-1; IL-6: interleukin-6; CRP: C-reactive protein; BMI: body mass index; kg: kilograms.

**Table 5:** Sensitivity analysis results for biomarkers, morphological variables, and physical performance in people with sarcopenic obesity.

Biomarkers							
	Excluded Studies	DFBETA	Cook’s Distance	DFFITS	ES (95%CI)	*p*	I^2^ (%)
IGF-1(ng/mL)	Chen, Chung, Chen, Ho, and Wu [22]	−2.34	0.95	−2.07	1.35 (1.01 to 1.69)	**<0.001**	0
IL-6 (pg/mL)	-	-	-	-	-	-	-
CRP (mg/L)	Dieli-Conwright, Courneya, Demark-Wahnefried, Sami, Lee, Buchanan, Spicer, Tripathy, Bernstein, and Mortimer [23]	10.01	1.93	6.30	−0.05 (−0.342 to 0.229)	0.69	1.56
Leptin (ng/mL)	Dieli-Conwright, Courneya, Demark-Wahnefried, Sami, Lee, Buchanan, Spicer, Tripathy, Bernstein, and Mortimer [23]	15.56	3.56	6.65	0.16 (−0.143 to 0.469)	0.29	0
Total cholesterol (mg/dL)	Dieli-Conwright, Courneya, Demark-Wahnefried, Sami, Lee, Buchanan, Spicer, Tripathy, Bernstein, and Mortimer [23]	2.38	0.90	2.38	0.02 (−0.537 to 0.540)	0.99	71.7
Triglycerides (mg/dL)	Dieli-Conwright, Courneya, Demark-Wahnefried, Sami, Lee, Buchanan, Spicer, Tripathy, Bernstein, and Mortimer [23]	13.66	2.99	6.47	−0.08 (−0.291 to 0.275)	0.95	0
**Morphological variables**							
BMI (kg/m^2^)	Ferhi, Gaied Chortane, Durand, Beaune, Boyas, and Maktouf [24]	0.97	0.33	0.87	0.41 (0.142 to 0.679)	**0.00**	0
Waist circumference (cm)	Dieli-Conwright, Courneya, Demark-Wahnefried, Sami, Lee, Buchanan, Spicer, Tripathy, Bernstein, and Mortimer [23]	1.66	0.75	1.71	0.81 (0.268 to 1.359)	**0.00**	60.4
Body fat (%)	-	-	-	-	-	-	-
ASM/weight (%)	-	-	-	-	-	-	-
Trunk fat (kg)	-	-	-	-	-	-	-
Body fat mass (kg)	Dieli-Conwright, Courneya, Demark-Wahnefried, Sami, Lee, Buchanan, Spicer, Tripathy, Bernstein, and Mortimer [23]	3.89	0.84	4.49	0.45 (0.043 to 0.862)	**0.03**	0
**Physical performance**							
Walking speed (m/s)	-	-	-	-	-	-	-
MIHS (kg)	Park, Kwon, and Park [28]	0.91	0.54	0.91	0.15 (−0.634 to 0.935)	0.707	90.5
Knee extension (kg)	Chen, Chung, Chen, Ho, and Wu [22] Vasconcelos, Dias, Araújo, Pinheiro, Moreira, and Dias [29]	0.83 1.60	0.54 1.08	0.83 1.43	0.48 (0.194 to 0.769)	**0.001**	4.39

**Table 6:** Results of the multivariate random-effect meta-regression for concurrent training variables to predict concurrent training in effects on morphological variables with body fat percentage in people with sarcopenic obesity.

Covariate	Coefficient	95% Cl	Z	*p*	R^2^
Body Fat (%) (n = 10)					
Intercept	−3.14	−26.8 to 20.5	−0.26	0.79	0.61
Weeks	0.13	0.02 to 0.24	2.30	**0.02**	0.32
Frequency of training	−0.06	−1.00 to 0.88	−0.13	0.89	−0.13
Minutes per session	0.06	−0.30 to 0.43	0.35	0.72	−0.11
Total, sessions	0.01	−0.01 to 0.03	0.83	0.40	−0.02

**Table 7:** GRADE assessment for the certainty of evidence.

Certainty Assessment							Number of Patients		Effect		Certainty	Importance
Number of Studies	Study Design	Risk of Bias	Inconsistency	Indirect evidence	Vagueness	Other Considerations	[Intervention]	[Comparison]	Relative (95% CI)	Absolute (95% CI)		
Biomarkers												
3	Randomized trials	Serious ^to^	It is not serious	It is not serious	It is not serious	None	148/288 (51.4%)	140/288 (48.6%)	Not estimable		Moderate	IMPORTANT
Morphological variables												
7	Randomized trials	Serious ^to^	It is not serious	It is not serious	It is not serious	None	237/460 (51.5%)	223/460 (48.5%)	Not estimable		Moderate	IMPORTANT
Physical performance												
5	Randomized trials	Serious ^to^	It is not serious	It is not serious	It is not serious	None	178/350 (50.9%)	172/350 (49.1%)	Not estimable		Moderate	IMPORTANT

## Data Availability

The datasets generated during and/or analyzed during the current research are as follows: available from the corresponding author upon reasonable request.
